# 4-Nitro­anilinium 3-carb­oxy-4-hy­droxy­benzene­sulfonate monohydrate

**DOI:** 10.1107/S1600536813026779

**Published:** 2013-10-05

**Authors:** P. K. Sivakumar, M. Krishna Kumar, G. Chakkaravarthi, R. Mohan Kumar, R. Kanagadurai

**Affiliations:** a Department of Physics, M. N. M. Jain Engineering College, Chennai 600 097, India; b Department of Physics, Presidency College, Chennai 600 005, India; c Department of Physics, CPCL Polytechnic College, Chennai 600 068, India

## Abstract

In the title hydrated salt, C_6_H_7_N_2_O_2_^+^·C_7_H_5_O_6_S^−^·H_2_O, the benzene ring of the cation makes a dihedral angle of 1.32 (19)° with the attached nitro group. In the anion, an intra­molecular O—H⋯O hydrogen bond with an *S*(6) ring motif is formed between the carb­oxyl and hy­droxy groups; the dihedral angle between the carb­oxyl group and the benzene ring is 8.76 (8)°. The crystal structure exhibits inter­molecular N—H⋯O, O—H⋯O, C—H⋯O, and π–π [centroid–centroid distances = 3.6634 (9) and 3.7426 (9) Å] inter­actions to form a three-dimensional network.

## Related literature

For mol­ecular compounds with nonlinear optical properties, see: Nalwa & Miyata (1997 ▶). For related structures, see: Asiri *et al.* (2010 ▶); Krishnakumar *et al.* (2012 ▶); Sudhahar *et al.* (2013 ▶).
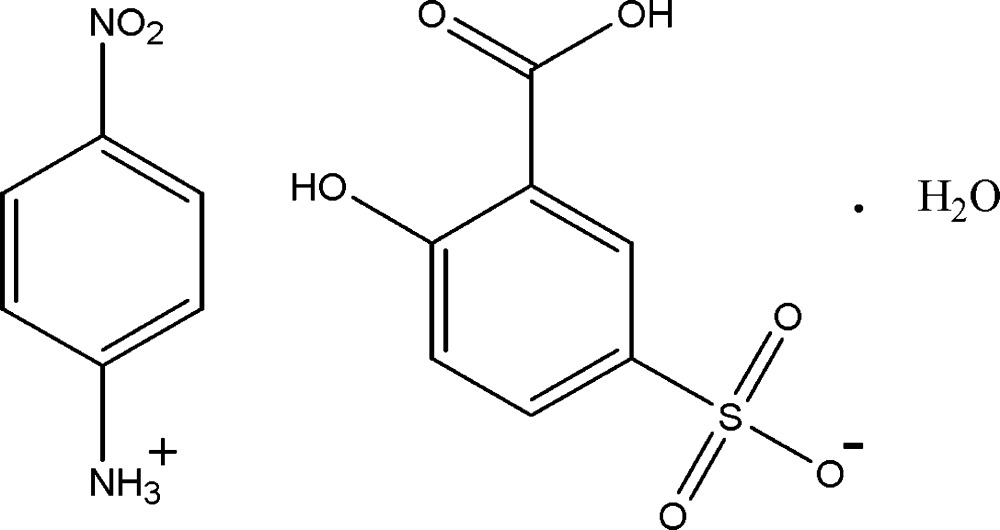


## Experimental

### 

#### Crystal data

C_6_H_7_N_2_O_2_^+^·C_7_H_5_O_6_S^−^·H_2_O
*M* *_r_* = 374.32
Orthorhombic, 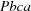

*a* = 13.2676 (3) Å
*b* = 13.5572 (3) Å
*c* = 17.1246 (4) Å
*V* = 3080.23 (12) Å^3^
*Z* = 8
Mo *K*α radiation
μ = 0.27 mm^−1^
*T* = 295 K
0.26 × 0.24 × 0.20 mm

#### Data collection

Bruker Kappa APEXII CCD diffractometer
Absorption correction: multi-scan (*SADABS*; Sheldrick, 1996 ▶) *T*_min_ = 0.934, *T*_max_ = 0.949
15578 measured reflections
3640 independent reflections
3062 reflections with *I* > 2σ(*I*)
*R*_int_ = 0.024

#### Refinement

*R*[*F* ^2^ > 2σ(*F* ^2^)] = 0.038
*wR*(*F* ^2^) = 0.107
*S* = 1.03
3640 reflections
236 parameters
2 restraints
H atoms treated by a mixture of independent and constrained refinement
Δρ_max_ = 0.44 e Å^−3^
Δρ_min_ = −0.34 e Å^−3^

### 

Data collection: *APEX2* (Bruker, 2004 ▶); cell refinement: *SAINT* (Bruker, 2004 ▶); data reduction: *SAINT*; program(s) used to solve structure: *SHELXS97* (Sheldrick, 2008 ▶); program(s) used to refine structure: *SHELXL97* (Sheldrick, 2008 ▶); molecular graphics: *PLATON* (Spek, 2009 ▶); software used to prepare material for publication: *SHELXL97*.

## Supplementary Material

Crystal structure: contains datablock(s) global, I. DOI: 10.1107/S1600536813026779/is5308sup1.cif

Structure factors: contains datablock(s) I. DOI: 10.1107/S1600536813026779/is5308Isup2.hkl

Supplementary material file. DOI: 10.1107/S1600536813026779/is5308Isup3.cml

Additional supplementary materials:  crystallographic information; 3D view; checkCIF report

## Figures and Tables

**Table 1:** Hydrogen-bond geometry (Å, °)

*D*—H⋯*A*	*D*—H	H⋯*A*	*D*⋯*A*	*D*—H⋯*A*
N1—H1*A*⋯O9	0.89	1.99	2.841 (2)	160
N1—H1*B*⋯O1	0.89	1.95	2.8357 (18)	171
O4—H4*A*⋯O5	0.82	1.88	2.6028 (18)	146
C9—H9⋯O9	0.93	2.57	3.141 (3)	121
N1—H1*A*⋯O7^i^	0.89	2.40	2.836 (2)	111
N1—H1*C*⋯O2^ii^	0.89	1.93	2.8069 (18)	168
O4—H4*A*⋯O2^iii^	0.82	2.38	2.9494 (16)	128
O6—H6⋯O3^iv^	0.82	1.86	2.6595 (17)	164
O9—H9*A*⋯O2^v^	0.82 (1)	2.33 (3)	3.005 (2)	139 (4)
O9—H9*A*⋯O3^v^	0.82 (1)	2.48 (3)	3.151 (2)	140 (4)
O9—H9*B*⋯O4^vi^	0.82 (1)	2.56 (3)	3.283 (2)	149 (4)

Symmetry codes: (i) 
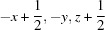
; (ii) 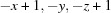; (iii) 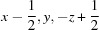; (iv) 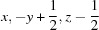; (v) 
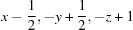
; (vi) 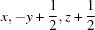.

**Fig. 1.**
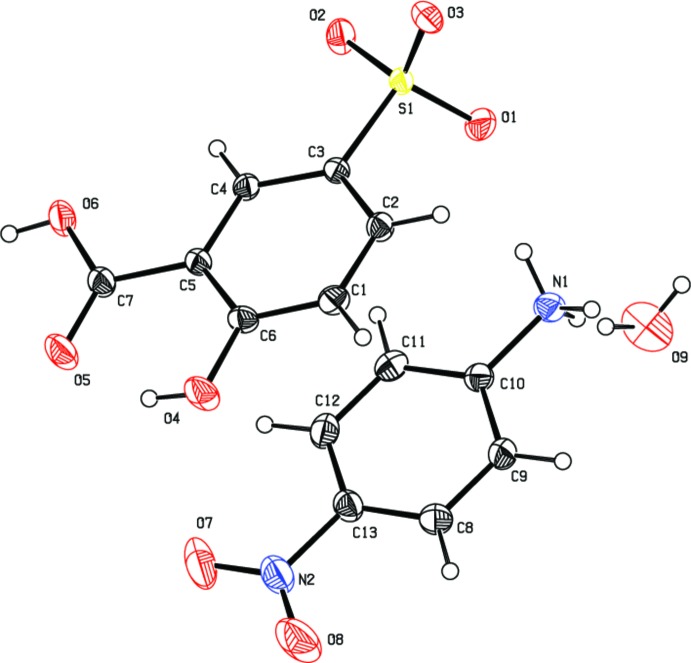
The molecular structure of the title compound, with atom labels and 30% probability displacement ellipsoids for non-H atoms.

**Fig. 2.**
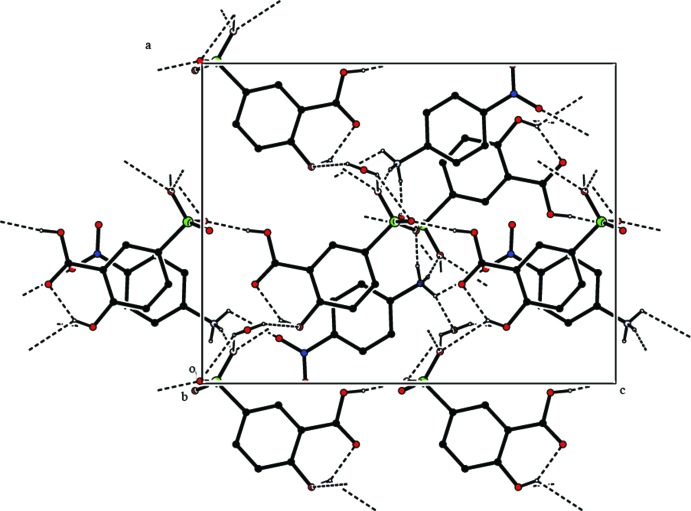
The packing of the title compound, viewed down the b axis. Intermolecular hydrogen bonds are shown as dashed lines. H atoms not involved in hydrogen bonding have been omitted.

## supplementary crystallographic information

### 1. Comment

In continuation of our studies of molecular compounds with non-linear optical properties which are used in optoelectronic and photonic devices (Nalwa & Miyata, 1997), we herewith report the crystal structure of the title compound (I), (Fig. 1). The title compound consists of one C_6_H_7_N_2_O_2_^+^ cation, one C_7_H_5_O_6_S^-^ anion and a water molecule in the asymmetric unit. The geometric parameters of the title compound are comparable with the reported structures (Asiri *et al.*, 2010; Krishnakumar *et al.*, 2012; Sudhahar *et al.*, 2013).

The dihedral angle between the two benzene rings (C1–C6) and (C8–C13) is 8.18 (7)°. The benzene ring (C1–C6) is planar [r.m.s. deviation = 0.0152 (17) Å] and makes a dihedral angle of 1.32 (19)° with the attached nitro group. The mean plane of carboxy group is inclined at an angle of 8.76 (8)° with the planar [r.m.s. deviation = 0.0137 (16) Å] benzene ring (C8–C13) of anion.

The crystal structure exhibits intermolecular N—H···O, O—H···O, C—H···O hydrogen bonds (Table 1 & Fig. 2) and π–π interactions [[*Cg*1···*Cg*2 = 3.7426 (9)Å, *Cg*1···*Cg*2^i^ = 3.6634 (9) Å; (i) 1/2-x, 1/2+y, z; *Cg*1 and *Cg*2 are the centroids of the rings (C1–C6) and (C8–C13), respectively] to form a three-dimensional molecular arrangement.

### 2. Experimental

The title compound was synthesized in ethanol by using 4-nitroaniline (6.90 g) and 5-sulfosalicylic acid dihydrate (12.711 g) in equimolar ratio. The saturated solution was allowed to evaporating slowly at room temperature. After the evaporation period of three weeks the crystals were collected and used for X-ray data collection.

### 3. Refinement

H atoms of the water molecule were located in a difference Fourier map and were refined; the O9—H9A and O9—H9B distances were restrained to 0.82 (1) Å. All other H atoms were positioned geometrically (C—H = 0.93 Å, N—H = 0.89 Å and O—H = 0.82 Å) and refined using riding model with *U*_iso_(H) = 1.2*U*_eq_(C) and 1.5*U*_eq_(N, O).

### Crystal data

**Table:**

C_6_H_7_N_2_O_2_^+^·C_7_H_5_O_6_S^−^·H_2_O	*F*(000) = 1552
*M**_r_* = 374.32	*D*_x_ = 1.614 Mg m^−^^3^
Orthorhombic, *P**b**c**a*	Mo *K*α radiation, λ = 0.71073 Å
Hall symbol: -P 2ac 2ab	Cell parameters from 3844 reflections
*a* = 13.2676 (3) Å	θ = 2.4–28.4°
*b* = 13.5572 (3) Å	µ = 0.27 mm^−^^1^
*c* = 17.1246 (4) Å	*T* = 295 K
*V* = 3080.23 (12) Å^3^	Block, colourless
*Z* = 8	0.26 × 0.24 × 0.20 mm

### Data collection

**Table:**

Bruker Kappa APEXII CCD diffractometer	3640 independent reflections
Radiation source: fine-focus sealed tube	3062 reflections with *I* > 2σ(*I*)
Graphite monochromator	*R*_int_ = 0.024
ω and φ scan	θ_max_ = 28.4°, θ_min_ = 2.4°
Absorption correction: multi-scan (*SADABS*; Sheldrick, 1996)	*h* = −15→11
*T*_min_ = 0.934, *T*_max_ = 0.949	*k* = −18→12
15578 measured reflections	*l* = −22→17

### Refinement

**Table:**

Refinement on *F*^2^	Secondary atom site location: difference Fourier map
Least-squares matrix: full	Hydrogen site location: inferred from neighbouring sites
*R*[*F*^2^ > 2σ(*F*^2^)] = 0.038	H atoms treated by a mixture of independent and constrained refinement
*wR*(*F*^2^) = 0.107	*w* = 1/[σ^2^(*F*_o_^2^) + (0.0571*P*)^2^ + 1.2353*P*] where *P* = (*F*_o_^2^ + 2*F*_c_^2^)/3
*S* = 1.03	(Δ/σ)_max_ < 0.001
3640 reflections	Δρ_max_ = 0.44 e Å^−^^3^
236 parameters	Δρ_min_ = −0.34 e Å^−^^3^
2 restraints	Extinction correction: *SHELXL97* (Sheldrick, 2008), Fc^*^=kFc[1+0.001xFc^2^λ^3^/sin(2θ)]^-1/4^
Primary atom site location: structure-invariant direct methods	Extinction coefficient: 0.0019 (4)

### Special details

**Table:**

Geometry. All esds (except the esd in the dihedral angle between two l.s. planes) are estimated using the full covariance matrix. The cell esds are taken into account individually in the estimation of esds in distances, angles and torsion angles; correlations between esds in cell parameters are only used when they are defined by crystal symmetry. An approximate (isotropic) treatment of cell esds is used for estimating esds involving l.s. planes.

### Fractional atomic coordinates and isotropic or equivalent isotropic displacement parameters (Å^2^)

**Table:**

	*x*	*y*	*z*	*U*_iso_*/*U*_eq_	
C1	0.23376 (11)	0.22732 (11)	0.36435 (9)	0.0324 (3)	
H1	0.1676	0.2374	0.3805	0.039*	
C2	0.31016 (11)	0.22696 (11)	0.41813 (9)	0.0291 (3)	
H2	0.2958	0.2364	0.4708	0.035*	
C3	0.41012 (10)	0.21236 (10)	0.39384 (8)	0.0249 (3)	
C4	0.43237 (10)	0.20070 (10)	0.31573 (8)	0.0267 (3)	
H4	0.4989	0.1928	0.2998	0.032*	
C5	0.35471 (11)	0.20079 (11)	0.26038 (8)	0.0277 (3)	
C6	0.25490 (11)	0.21266 (11)	0.28527 (9)	0.0304 (3)	
C7	0.37580 (12)	0.18773 (12)	0.17625 (9)	0.0343 (3)	
C8	0.10072 (13)	−0.02919 (13)	0.38844 (10)	0.0402 (4)	
H8	0.0307	−0.0320	0.3888	0.048*	
C9	0.15443 (12)	−0.02301 (13)	0.45736 (9)	0.0371 (4)	
H9	0.1210	−0.0220	0.5051	0.045*	
C10	0.25815 (12)	−0.01838 (11)	0.45470 (9)	0.0301 (3)	
C11	0.31058 (12)	−0.02335 (11)	0.38520 (10)	0.0359 (4)	
H11	0.3807	−0.0228	0.3848	0.043*	
C12	0.25710 (13)	−0.02920 (12)	0.31634 (10)	0.0391 (4)	
H12	0.2905	−0.0319	0.2686	0.047*	
C13	0.15317 (13)	−0.03101 (12)	0.31948 (9)	0.0361 (4)	
N1	0.31299 (10)	−0.00774 (10)	0.52813 (8)	0.0351 (3)	
H1A	0.2733	0.0208	0.5634	0.053*	
H1B	0.3674	0.0294	0.5205	0.053*	
H1C	0.3317	−0.0670	0.5452	0.053*	
N2	0.09540 (14)	−0.03548 (12)	0.24631 (9)	0.0513 (4)	
O1	0.47752 (9)	0.12506 (9)	0.51725 (7)	0.0434 (3)	
O2	0.59937 (8)	0.18656 (10)	0.42481 (7)	0.0403 (3)	
O3	0.50743 (9)	0.30034 (8)	0.50523 (7)	0.0391 (3)	
O4	0.17634 (9)	0.21175 (11)	0.23553 (7)	0.0485 (4)	
H4A	0.1969	0.2027	0.1909	0.073*	
O5	0.30993 (9)	0.17648 (13)	0.12778 (7)	0.0580 (4)	
O6	0.47157 (9)	0.18949 (11)	0.15782 (7)	0.0489 (3)	
H6	0.4778	0.1818	0.1106	0.073*	
O7	0.14092 (17)	−0.03731 (18)	0.18586 (9)	0.1005 (8)	
O8	0.00426 (13)	−0.03699 (16)	0.24947 (10)	0.0834 (6)	
O9	0.16700 (15)	0.10629 (14)	0.60925 (13)	0.0740 (5)	
S1	0.50511 (2)	0.20521 (3)	0.46557 (2)	0.02593 (12)	
H9A	0.152 (3)	0.149 (2)	0.5775 (18)	0.143 (16)*	
H9B	0.186 (3)	0.136 (3)	0.6481 (16)	0.167 (19)*	

### Atomic displacement parameters (Å^2^)

**Table:**

	*U*^11^	*U*^22^	*U*^33^	*U*^12^	*U*^13^	*U*^23^
C1	0.0208 (7)	0.0399 (8)	0.0365 (8)	0.0032 (6)	0.0027 (6)	−0.0022 (6)
C2	0.0273 (7)	0.0328 (7)	0.0273 (7)	0.0014 (6)	0.0032 (6)	−0.0012 (6)
C3	0.0228 (7)	0.0278 (7)	0.0240 (7)	−0.0001 (5)	−0.0020 (5)	0.0014 (5)
C4	0.0208 (6)	0.0333 (7)	0.0260 (7)	0.0000 (5)	0.0008 (5)	0.0018 (6)
C5	0.0250 (7)	0.0336 (7)	0.0245 (7)	−0.0008 (6)	−0.0022 (5)	0.0009 (6)
C6	0.0227 (7)	0.0364 (8)	0.0323 (8)	0.0010 (6)	−0.0050 (6)	0.0002 (6)
C7	0.0281 (8)	0.0483 (9)	0.0266 (7)	−0.0008 (6)	−0.0025 (6)	0.0017 (7)
C8	0.0297 (8)	0.0515 (10)	0.0396 (9)	−0.0019 (7)	−0.0032 (7)	0.0014 (7)
C9	0.0310 (8)	0.0503 (10)	0.0301 (8)	−0.0037 (7)	0.0030 (6)	0.0015 (7)
C10	0.0304 (8)	0.0277 (7)	0.0322 (7)	−0.0013 (6)	−0.0031 (6)	0.0043 (6)
C11	0.0295 (8)	0.0346 (8)	0.0436 (9)	−0.0011 (6)	0.0052 (7)	0.0011 (7)
C12	0.0455 (9)	0.0387 (8)	0.0332 (8)	−0.0023 (7)	0.0086 (7)	0.0014 (7)
C13	0.0426 (9)	0.0348 (8)	0.0307 (8)	−0.0011 (7)	−0.0049 (7)	0.0024 (6)
N1	0.0306 (7)	0.0385 (7)	0.0362 (7)	−0.0016 (5)	−0.0047 (5)	0.0027 (6)
N2	0.0630 (11)	0.0556 (10)	0.0354 (8)	−0.0016 (8)	−0.0099 (7)	0.0018 (7)
O1	0.0402 (6)	0.0507 (7)	0.0394 (6)	−0.0103 (5)	−0.0074 (5)	0.0197 (6)
O2	0.0242 (5)	0.0657 (8)	0.0310 (6)	0.0081 (5)	0.0021 (4)	0.0055 (5)
O3	0.0451 (7)	0.0419 (7)	0.0302 (6)	−0.0013 (5)	−0.0072 (5)	−0.0047 (5)
O4	0.0238 (6)	0.0848 (10)	0.0370 (7)	0.0052 (6)	−0.0083 (5)	−0.0074 (6)
O5	0.0323 (7)	0.1114 (12)	0.0304 (6)	−0.0045 (7)	−0.0071 (5)	−0.0088 (7)
O6	0.0289 (6)	0.0934 (10)	0.0243 (6)	−0.0025 (6)	0.0010 (5)	0.0018 (6)
O7	0.0957 (14)	0.176 (2)	0.0304 (8)	−0.0207 (14)	−0.0011 (8)	0.0029 (10)
O8	0.0567 (11)	0.1344 (19)	0.0590 (10)	0.0062 (10)	−0.0251 (8)	−0.0088 (11)
O9	0.0787 (12)	0.0606 (10)	0.0827 (13)	0.0281 (9)	−0.0161 (10)	−0.0100 (10)
S1	0.0221 (2)	0.0348 (2)	0.02094 (19)	−0.00090 (13)	−0.00073 (12)	0.00349 (13)

### Geometric parameters (Å, º)

**Table:**

C1—C2	1.370 (2)	C10—C11	1.380 (2)
C1—C6	1.397 (2)	C10—N1	1.4599 (19)
C1—H1	0.9300	C11—C12	1.378 (2)
C2—C3	1.4040 (19)	C11—H11	0.9300
C2—H2	0.9300	C12—C13	1.380 (2)
C3—C4	1.379 (2)	C12—H12	0.9300
C3—S1	1.7625 (14)	C13—N2	1.470 (2)
C4—C5	1.400 (2)	N1—H1A	0.8900
C4—H4	0.9300	N1—H1B	0.8900
C5—C6	1.400 (2)	N1—H1C	0.8900
C5—C7	1.478 (2)	N2—O7	1.199 (2)
C6—O4	1.3462 (18)	N2—O8	1.211 (2)
C7—O5	1.2150 (19)	O1—S1	1.4484 (12)
C7—O6	1.309 (2)	O2—S1	1.4544 (11)
C8—C13	1.371 (2)	O3—S1	1.4579 (12)
C8—C9	1.381 (2)	O4—H4A	0.8200
C8—H8	0.9300	O6—H6	0.8200
C9—C10	1.378 (2)	O9—H9A	0.821 (10)
C9—H9	0.9300	O9—H9B	0.815 (10)
			
C2—C1—C6	120.18 (13)	C11—C10—N1	119.75 (14)
C2—C1—H1	119.9	C12—C11—C10	118.75 (15)
C6—C1—H1	119.9	C12—C11—H11	120.6
C1—C2—C3	120.03 (13)	C10—C11—H11	120.6
C1—C2—H2	120.0	C11—C12—C13	118.81 (15)
C3—C2—H2	120.0	C11—C12—H12	120.6
C4—C3—C2	120.39 (13)	C13—C12—H12	120.6
C4—C3—S1	121.11 (11)	C8—C13—C12	122.71 (16)
C2—C3—S1	118.47 (11)	C8—C13—N2	118.05 (16)
C3—C4—C5	119.94 (13)	C12—C13—N2	119.24 (16)
C3—C4—H4	120.0	C10—N1—H1A	109.5
C5—C4—H4	120.0	C10—N1—H1B	109.5
C4—C5—C6	119.34 (13)	H1A—N1—H1B	109.5
C4—C5—C7	121.36 (13)	C10—N1—H1C	109.5
C6—C5—C7	119.30 (13)	H1A—N1—H1C	109.5
O4—C6—C1	117.34 (13)	H1B—N1—H1C	109.5
O4—C6—C5	122.58 (14)	O7—N2—O8	122.79 (19)
C1—C6—C5	120.08 (13)	O7—N2—C13	118.31 (19)
O5—C7—O6	122.38 (15)	O8—N2—C13	118.91 (17)
O5—C7—C5	123.01 (15)	C6—O4—H4A	109.5
O6—C7—C5	114.61 (13)	C7—O6—H6	109.5
C13—C8—C9	118.38 (16)	H9A—O9—H9B	106 (4)
C13—C8—H8	120.8	O1—S1—O2	112.34 (7)
C9—C8—H8	120.8	O1—S1—O3	112.60 (8)
C10—C9—C8	119.32 (15)	O2—S1—O3	111.05 (7)
C10—C9—H9	120.3	O1—S1—C3	106.64 (7)
C8—C9—H9	120.3	O2—S1—C3	106.86 (7)
C9—C10—C11	121.97 (15)	O3—S1—C3	106.93 (7)
C9—C10—N1	118.28 (14)		
			
C6—C1—C2—C3	0.3 (2)	C8—C9—C10—N1	177.48 (15)
C1—C2—C3—C4	1.6 (2)	C9—C10—C11—C12	2.6 (2)
C1—C2—C3—S1	−176.32 (11)	N1—C10—C11—C12	−177.29 (14)
C2—C3—C4—C5	−1.7 (2)	C10—C11—C12—C13	−0.7 (2)
S1—C3—C4—C5	176.15 (10)	C9—C8—C13—C12	1.5 (3)
C3—C4—C5—C6	−0.1 (2)	C9—C8—C13—N2	−178.74 (15)
C3—C4—C5—C7	−179.54 (14)	C11—C12—C13—C8	−1.3 (3)
C2—C1—C6—O4	178.63 (14)	C11—C12—C13—N2	178.93 (14)
C2—C1—C6—C5	−2.1 (2)	C8—C13—N2—O7	−179.8 (2)
C4—C5—C6—O4	−178.81 (14)	C12—C13—N2—O7	−0.1 (3)
C7—C5—C6—O4	0.7 (2)	C8—C13—N2—O8	0.3 (3)
C4—C5—C6—C1	2.0 (2)	C12—C13—N2—O8	−179.96 (18)
C7—C5—C6—C1	−178.55 (14)	C4—C3—S1—O1	−120.21 (13)
C4—C5—C7—O5	171.57 (17)	C2—C3—S1—O1	57.67 (13)
C6—C5—C7—O5	−7.9 (3)	C4—C3—S1—O2	0.12 (14)
C4—C5—C7—O6	−8.6 (2)	C2—C3—S1—O2	178.00 (11)
C6—C5—C7—O6	171.95 (14)	C4—C3—S1—O3	119.10 (12)
C13—C8—C9—C10	0.3 (3)	C2—C3—S1—O3	−63.02 (13)
C8—C9—C10—C11	−2.4 (3)		

### Hydrogen-bond geometry (Å, º)

**Table:**

*D*—H···*A*	*D*—H	H···*A*	*D*···*A*	*D*—H···*A*
N1—H1*A*···O9	0.89	1.99	2.841 (2)	160
N1—H1*B*···O1	0.89	1.95	2.8357 (18)	171
O4—H4*A*···O5	0.82	1.88	2.6028 (18)	146
C9—H9···O9	0.93	2.57	3.141 (3)	121
N1—H1*A*···O7^i^	0.89	2.40	2.836 (2)	111
N1—H1*C*···O2^ii^	0.89	1.93	2.8069 (18)	168
O4—H4*A*···O2^iii^	0.82	2.38	2.9494 (16)	128
O6—H6···O3^iv^	0.82	1.86	2.6595 (17)	164
O9—H9*A*···O2^v^	0.82 (1)	2.33 (3)	3.005 (2)	139 (4)
O9—H9*A*···O3^v^	0.82 (1)	2.48 (3)	3.151 (2)	140 (4)
O9—H9*B*···O4^vi^	0.82 (1)	2.56 (3)	3.283 (2)	149 (4)

Symmetry codes: (i) −*x*+1/2, −*y*, *z*+1/2; (ii) −*x*+1, −*y*, −*z*+1; (iii) *x*−1/2, *y*, −*z*+1/2; (iv) *x*, −*y*+1/2, *z*−1/2; (v) *x*−1/2, −*y*+1/2, −*z*+1; (vi) *x*, −*y*+1/2, *z*+1/2.
